# Comparing caloric restriction regimens for effective weight management in adults: a systematic review and network meta-analysis

**DOI:** 10.1186/s12966-024-01657-9

**Published:** 2024-09-26

**Authors:** Jinming Huang, Yi Li, Maohua Chen, Zhaolun Cai, Zhen Cai, Zhiyuan Jiang

**Affiliations:** 1 Department of Rehabilitation Medicine, Key Laboratory of Rehabilitation Medicine, West China Hospital, Sichuan University, Chengdu, China; 2 Department of Plastic Surgery, Sichuan Provincial People’s Hospital, University of Electronic Science and Technology of China, Chengdu, Sichuan China; 3 Department of Gastrointestinal Surgery, West China Hospital, Sichuan University, Chengdu, Sichuan China

**Keywords:** Body weight, Intermittent fasting, Calorie restriction, Alternate day fasting, Time restrict eating, Short-term fasting

## Abstract

**Background:**

Randomized controlled trials have confirmed the effectiveness of four prevalent caloric restriction regimens in reducing obesity-related health risks. However, there is no consensus on the optimal regimen for weight management in adults.

**Methods:**

We systematically searched PubMed, Embase, Web of Science, and Cochrane CENTRAL up to January 15, 2024, for randomized controlled trials (RCT) involving adults, evaluating the weight-loss effects of alternate day fasting (ADF), short-term fasting (STF), time-restricted eating (TRE), and continuous energy restriction (CER). The primary outcome was body weight, with secondary outcomes including BMI, fat mass, lean mass, waist circumference, fasting glucose, HOMA-IR, and adverse events. Bayesian network meta-analysis was conducted, ranking regimens using the surface under the cumulative ranking curve and the probability of being the best. Study quality was assessed using the Confidence in Network Meta-Analysis tool.

**Results:**

Data from 47 RCTs (representing 3363 participants) were included. ADF showed the most significant body weight loss (Mean difference (MD): -3.42; 95% Confidence interval (CI): -4.28 to -2.55), followed by TRE (MD: -2.25; 95% CI: -2.92 to -1.59). STF (MD: -1.87; 95% CI: -3.32 to -0.56) and CER (MD: -1.59; 95% CI: -2.42 to -0.79) rank third and fourth, respectively. STF lead to decline in lean mass (MD: -1.26; 95% CI: -2.16, -0.47). TRE showed benefits on fasting glucose (MD: -2.98; 95% CI: -4.7, -1.26). Subgroup analysis revealed all four caloric restriction regimens likely lead to modest weight loss after 1–3 months, with ADF ranked highest, but by 4–6 months, varying degrees of weight regain occur, particularly with CER, while interventions lasting 7–12 months may result in effective weight loss, with TRE potentially ranking first during both the 4–6 months and 7–12 months periods. ADF showing fewer and shorter-lasting physical symptoms.

**Conclusion:**

All four included regiments were effective in reducing body weight, with ADF likely having the most significant impact. Each regimen likely leads to modest weight loss after 1–3 months, followed by weight regain by 4–6 months. However, interventions lasting 7–12 months achieve greater weight loss overall.

**Trial registration:**

PROSPERO: CRD42022382478.

## Background

Overweight and obesity significantly elevate the risk of non-communicable diseases (NCDs) [1, 2], encompassing cardiovascular diseases (such as hypertension, myocardial infarction, and stroke) [3, 4], metabolic diseases (including type 2 diabetes mellitus and fatty liver disease) [5], musculoskeletal ailments (like osteoarthritis) [6], and digestive system cancers (comprising esophagus, stomach, pancreas, liver, and colon) [7–9]. These NCDs account for more than 70% of premature deaths worldwide and stand as a primary cause of disability [10]. Over the past four decades, obesity prevalence has doubled in over 70 countries and continues to escalate in most others [11]. Projections from the World Obesity Federation indicate that without enhancements in prevention and treatment, over 4 billion individuals (equivalent to 51% of the global population) will grapple with overweight or obesity by 2035, resulting in an annual global economic burden of $4.32 trillion [12]. Fortunately, weight loss interventions have demonstrated efficacy in reducing blood pressure, glucose levels, blood lipids, and the risk of both cardiovascular disease and all-cause mortality [13].

Lifestyle interventions, encompassing caloric restriction, physical activity, and behavioral therapy, constitute cornerstones in the clinical management of obesity [14]. Pharmacotherapy or bariatric surgery is recommended for individuals inadequately responsive to these lifestyle interventions [14, 15]. Caloric restrictions are integral facets of these lifestyle interventions and can be classified into four common regimens [16]: Continuous Energy Restriction (CER, entailing a daily energy intake reduction of 20–30% from daily requirements), short-term fasting (STF, involving limiting daily energy intake to approximately 25% on either 2–3 consecutive or non-consecutive days of the week), Alternate Day Fasting (ADF, comprising consuming 20–30% of daily energy needs on fasting days and consuming 100% of daily energy needs or ad libitum on non-fasting days), and Time-Restricted Eating (TRE, characterized by consuming within a daily window of less than 12 h). These latter three regimens collectively fall under the umbrella of Intermittent Energy Restriction (IER).

The optimal regimen for weight loss remains uncertain. An umbrella review of meta-analyses favored IER, particularly the ADF regimen, for overweight or obese adults [17]. Another meta-analysis suggested that the weight loss effects of IER were comparable to CER [18]. Conversely, Templeman et al. [19] found that the ADF regimen was less effective in reducing body fat mass compared to matched CER. Lowe et al. [20], in a three-week randomized controlled trial (RCT), observed the effectiveness of the TRE program for weight loss, albeit with lean mass loss instead of fat mass. Laurens et al. [21] reported that protein loss primarily occurred in the early stages of the CER regimen but diminished as ketogenesis increased.

Identifying the most effective, low-side-effect caloric restriction strategy holds promise for reducing the incidence of NCDs and alleviating the associated healthcare burden. To date, existing systematic reviews and meta-analyses have conducted pairwise comparisons, such as STF versus CER [22], IER versus CER [23], and STF versus CER [24, 25]. However, these studies have not yielded conclusive evidence regarding the optimal regimen. To address these gaps, we conducted a network meta-analysis that estimates the relative effects of the four commonly used caloric restriction strategies by incorporating both direct comparisons (from head-to-head trials) and indirect comparisons (through a common comparator). Additionally, we noticed variations in outcome measures across different intervention durations in RCTs. Therefore, we performed subgroup analyses based on intervention duration. This study aims to elucidate the effectiveness, sustainability, and safety of these calorie restriction regimens, helping healthcare professionals and individuals seeking weight loss to make more informed decisions.

## Methods

This study adhered to the PRISMA for Network Meta-Analyses (PRISMA-NMA) guidelines [26] and followed the standard methodology recommended by the Cochrane Collaboration [27]. The study protocol was prospectively registered in the International Prospective Register of Systematic Reviews (PROSPERO) under the code CRD42022382478.

### Inclusion criteria

We applied the PICOS model [28] (Population, Intervention, Comparator, Outcome, and Study Design) to define the inclusion criteria: (1) Population: Adults ≥ 18 years with stable weight (loss or gain < 5 kg in the past 3 months). (2) Interventions: Evaluated CER, STF, ADF, and TRE, with no calorie restriction. (3) Comparisons: General diet without time or calorie restrictions. (4) Outcomes: Primary outcome: changes in body weight (kg); Secondary outcomes: BMI (kg/m^2^), fat mass (kg), lean mass (kg), waist circumference (cm), fasting glucose (mg/dL), Homeostatic Model Assessment of Insulin Resistance (HOMA-IR), and adverse events. (5) Study design: Published RCTs with ≥ 4 weeks intervention, no language or publication year restrictions.

### Exclusion criteria

(1) Use of weight-loss medications/supplements, surgeries, pregnancy, lactation, significant endocrinopathies (e.g., diabetes, thyroid disease), cancer, chronic diseases needing dietary control (e.g., heart, and liver disease, chronic nephrosis, chronic diarrheal disease), steroids, or immunosuppressants within 3 months. (2) Excluded studies < 4 weeks, religious fasts (e.g., Muslim Ramadan fasting and Greek Orthodox fasting), and those limiting dietary components (e.g., low-carbohydrate diets). (3) Excluded inaccessible full-text articles or studies with overlapping data, even after reasonable attempts to contact corresponding authors.

### Literature search

We conducted a comprehensive literature search to identify relevant RCTs published from the inception of the databases up to January 15, 2024. This search utilized a combination of four databases: PubMed, Embase (Ovid), Web of Science, and the Cochrane Central Register of Controlled Trials (CENTRAL), using terms such as "intermittent fasting," "calorie restriction," "time-restricted eating," "alternate day fasting," and their derivatives. A detailed list of the search strategy is available in Supplemental Material 1. Following the database search, Three independent reviewers screened titles and abstracts, and conducted a full-text assessment of relevant articles, resolving any disagreements through discussion. two of these reviewers then examined reference lists of included studies to identify additional relevant studies.

### Quality assessment

Two reviewers independently assessed the risk of bias of the included studies using the Cochrane Handbook’s Risk of Bias Assessment Tool [29] and resolved disagreements through consultation and discussion with a third reviewer. Publication bias was assessed using funnel plots. The Confidence in Network Meta-Analysis (CINeMA) tool [30, 31] was employed to assess the quality of evidence in six domains, including within-study bias, across-studies bias, indirectness, imprecision, heterogeneity, and incoherence.

### Data extraction

Data extraction underwent a rigorous process with two independent reviewers employing a standardized electronic form. Oversight by a third author ensured statistical rigor. Extracted data included publication year, study period, baseline population characteristics, intervention specifics, duration of intervention and follow-up, relative study quality parameters, and primary and secondary outcomes (mean and standard deviation [SD] of change scores between post-intervention and baseline measurements). In cases where multiple studies reported results from the same subjects, inclusion prioritization followed guidelines favoring the study with the most comprehensive data, the most recent publication date, or the highest methodological quality based on the Cochrane Risk of Bias Assessment Tool. Finally, we assessed the network geometry by constructing diagrams that visually represented direct comparisons between interventions, with node sizes and edge thicknesses indicating the number and distribution of studies.

### Data synthesis and statistical analysis

Network meta-analysis was conducted using R 4.2.2 software with the 'gemtc' package in RStudio, employing Bayesian frameworks alongside Markov Chain Monte Carlo simulations. The estimation process included 25,000 burn-in iterations and 50,000 sampling iterations across four chains initialized with different values. Model selection was based on the deviance information criterion, with a significant difference in consistency test results considered when the deviance information criterion exceeded 5. Next, we calculated intervention rankings using the Surface Under the Cumulative Ranking Curve (SUCRA) and the Probability of Being the Best (Prbest) in R 4.2.2. Higher SUCRA and Prbest scores indicate a greater likelihood of a regimen occupying a top position [26]. Heterogeneity was assessed using Cochran's Q statistic and *I*^*2*^ values, classifying *I*^*2*^ < 50% as low heterogeneity and *I*^*2*^ ≥ 50% as high heterogeneity.

Network plots were generated in CINeMA framework and STATA 15.0 to visualize intervention relationships, with node size reflecting group sample sizes and edge width representing the number of studies.

Subsequently, data were categorized into 1–3 month, 4–6 month, and 7–12 month periods for subgroup analysis, and the above steps were repeated.

## Results

### Study selection

**Fig. 1:**
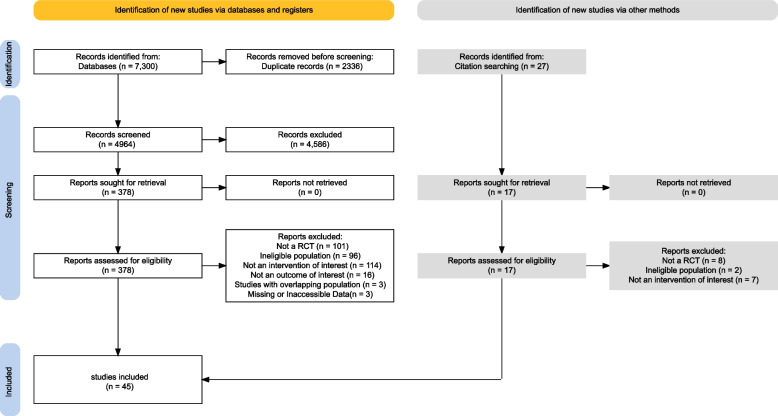
PRISMA flowchart

Initially, a total of 7300 potential records were identified through database searches. Furthermore, an additional 27 potentially eligible records were extracted from the reference lists of relevant reviews. This process culminated in the comprehensive evaluation of 395 full-text articles for eligibility. Subsequently, 47 RCTs [20, 32–77] met the inclusion criteria and were integrated into this systematic review and quantitative synthesis network meta-analysis, as depicted in Fig. 1 (PRISMA Flow Diagram).

### Study characteristics

We included 47 articles involving a total of 3363 participants (Table 1). All studies were conducted as single-center RCTs. Among these participants, 69.04% were female, and over 58.49% were classified as overweight or obese. The distribution of studies among the four caloric intervention regimens was as follows: 24 for CER, 24 for TRE, 15 for ADF, and nine for STF. Regarding the duration of interventions, 40 studies reported results for the 1–3 month period, eight reported outcomes for the 4–6 month period, and six reported outcomes for the 7–12 month period. Furthermore, four studies incorporated individuals engaged in regular exercise [33, 58, 72, 73], and an additional three studies involved participants with insulin resistance but without a diabetes diagnosis [38, 45, 63].

**Table 1:** Characteristics of studies

Author, year	Country	Trial ID	N	Period	Study design	Comparison	Age (year)	Female (%)	BMI (kg/m^2^)	Body fat percentage (%)	Follow up, dropout rate (%)
Liu, 2023 [56]	China	NA	80	September 2021 to April 2022	RCT, single center, 4 arms, parallel	General diet vs. TRE vs. Exercise (excluded) vs. TRE + Exercise (excluded)	18–22	100	18.5–23.9	≥ 30	2 months, 5
Irani, 2023 [52]	Iran	IRCT20131228015968N7	89	October 2020 to December 2021	RCT, single center, 2 arms, parallel	TRE vs. CER	20–65	100	26–40	≥ 35	2 months, 37.08
Hooshiar, 2023 [50]	Iran	IRCT20220522054958N3	56	NA	RCT, single center, 2 arms, parallel	ADF vs. CER	18–50	100	25–40	NA	2 months,16.07
Fagundes, 2023 [44]	Brazil	NCT03574103, CAAE72774617.6.0000.5149	36	NA	RCT, single center, 2 arms, parallel	TRE vs. CER	18–59	100	≥ 25	≥ 35	1 month, 36.11 2 months, 44.44
Domaszewski, 2022 [43]	Poland	NA	46	NA	RCT, single center, 2 arms, parallel	TRE vs. General diet	65–74	0	25–29.9	20–35	1.5 month, 0
Jamshed, 2022 [53]	USA	NCT03459703	90	August 2018 to April 2020	RCT, single center, 2 arms, parallel	TRE vs. CER	25–75	80	28–45	NA	3.5 months, 34.44
Liu, 2022 [55]	China	NCT03745612	139	November 2018 to July 2021	RCT, single center, 2 arms, parallel	TRE vs. CER	18–75	48.92	28–45	≥ 30	6 months, 0 12 months, 0
Thomas, 2022 [71]	USA	NCT03571048	85	July 2018 to February 2020	RCT, single center, 2 arms, parallel	TRE vs. CER	18–50	85.19	27–45	≥ 35	3 months, 17.65 10 months, 25.88
Xie, 2022 [76]	China	ChiCTR2000029797	82	February 2020, to March 2020	RCT, single center, 3 arms, parallel	eTRE vs. mTRE vs. General diet	18–45	78.04	17.5–30.0	NA	5 weeks, 0
Queiroz, 2022 [65]	Brazil	NCT04647149	48	September 2019 to May 2020	RCT, single center, 3 arms, parallel	early TRE vs. delayed TRE vs. CER	20–40	87.5	25.0 -34.9	≥ 35	2 months, 22.92
Zhang, 2022 [24, 77]	China	ChiCTR2000039115	63	NA	RCT, single center, 3 arms, parallel	early TRE vs. delayed TRE vs. General diet	18–30	45	≥ 24	≥ 30	2 months, 4.76
Kotarsky, 2021 [54]	USA	NCT03823872	23	October 2018 to December 2019	RCT, single center, 2 arms, parallel	TRE vs. General diet	35–60	85.7	25.0–34.9	≥ 35	2 months, 8.70
Pureza, 2021 [41]	Brazil	RBR-387v6v	58	July 2018 to November 2019	RCT, single center, 2 arms, parallel	TRE vs. CER	19–44	100	30–45	≥ 35	12 months, 53.45
Phillips, 2021 [63]	Switzerland	NCT03241121 and SNCTP000002259	54	NA	RCT, single center, 2 arms, parallel	TRE vs. General diet	≥ 18	71.6	≥ 30	NA	6 months, 24.07
Brady, 2021 [33]	Ireland	NA	23	NA	RCT, single center, 2 arms, parallel	TRE vs. General diet	29–44	0	NA	NA	2 months, 26.09
Steger, 2021 [67]	USA	NCT03696966	35	June 2017 to October 2018	RCT, single center, 2 arms, parallel	ADF vs. CER	21–65	77.14	25–35	NA	3 months, 0 6 months, 0
Lowe, 2020 [20]	USA	NCT03393195 and NCT03637855	46	August 2018 to June 2019	RCT, single center, 2 arms, parallel	TRE vs. General diet	18–64	44.0	27–43	NA	3 months, 0
Cienfuegos, 2020 [38]	USA	NCT03867773	54	February 2019 to October 2019	RCT, single center, 3 arms, parallel	TRE(20 h) vs. TRE(18 h) vs. General diet	18–65	93.10	30–50	≥ 35	2 months, 9.26
Martens, 2020 [57]	USA	NCT02970188	24	June 2016 to December 2017	RCT, single center, 2 arms, crossover	TRE vs. General diet	55–79	54.5	average 24.7	≥ 25	6 weeks, 8.33
Pinto, 2020 [64]	UK	NCT02679989	45	February to July 2016	RCT, single center, 2 arms, parallel	STF vs. CER	35–75	72.09	average 31	≥ 30	1 month, 4.44
Domaszewski, 2020 [42]	Poland	NA	45	NA	RCT, single center, 2 arms, parallel	TRE vs. General diet	≥ 60	100	≥ 25	NA	1.5 month, 6.66
Chow, 2020 [37]	USA	NCT03129581	22	NA	RCT, single center, 2 arms, parallel	TRE vs. General diet	18–65	85	≥ 25	≥ 30	3 months, 9.09
Tinsley, 2019^a ^ [73]	USA	NCT03404271	40	January to August 2018	RCT, single center, 3 arms, parallel	TRE vs. TRE plus 3 g/d HMB (excluded) vs. General diet	18–30	100	NA	15–29	2 months,40.00
Stekovic, 2019 [68]	Germany	NCT02673515	60	NA	a prospective cohort study(excluded) with an embedded RCT, single center, 2 arms, parallel	ADF vs. General diet	35–65	NA	22–30	NA	1 months, 5.00
Parvaresh, 2019 [62]	Iran	IRCT201509092395N8	70	December 2015 to March 2016	RCT, single center, 2 arms, parallel	ADF vs. CER	25–60	40.0	25–40	≥ 30	2 months, 1.43
Panizza, 2019 [61]	USA	NCT03639350	60	September 2016 to October 2017	RCT, single center, 2 arms, parallel	STF vs. General diet	35–55	70.0	25–40	≥ 30	3 months, 20.00
Hutchison, 2019 [51]	Australia	NCT01769976	88	March 2013 to September 2015	RCT, single center, 4 arms, parallel	ADF- energy restriction vs. ADF vs. CER vs. General diet	35–70	88	25–42	≥ 40	2 months, 11.36
Gabel, 2019 [45]	USA	NCT00960505	53	October 2011 to January 2015	RCT, single center, 3 arms, parallel	ADF vs. CER vs. General diet	18–65	76	25–39.9	NA	6 months, NA 12 months, 18.87
Cho, 2019 [36]	South Korea	NCT03652532	48	April 2014 to March 2016	RCT, single center, 4 arms, parallel	ADF vs. General diet vs. Exercise(excluded) vs. ADF + Exercise (excluded)	20–65	NA	> 23.0	≥ 25	2 months, 27.08
Cai, 2019 [34]	China	ChiCTR1900024411	271	2018	RCT, single center, 3 arms, parallel	ADF vs. TRE vs. CER	18–65	75	> 24	NA	3 months, 2.58
Hirsh, 2019 [49]	USA	NCT03372109	23	November 2017 to January 2018	RCT, single center, 2 arms, parallel	STF vs. General diet	21–65	60.87	25–29·9	NA	1.5 month, 4.34
Headland, 2019 [48]	Australia	ACTRN12614001041640	332	August 2014 to September 2016	RCT, single center, 3 arms, parallel	STF vs. CER vs. week-on-week-off energy restriction (excluded)	18–72	83.13	≥ 27	≥ 40	2 months, 26.51 12 months, 56.02
Coutinho, 2018 [40]	Norway	NCT02169778	35	NA	RCT, single center, 2 arms, parallel	ADF vs. CER	18–65	78.57	30–40	≥ 35	3 month, 20.0
Sundfor, 2018 [70]	Norway	NCT02480504	112	August 2015 to April 2017	RCT, single center, 2 arms, parallel	STF vs. CER	21–70	50.0	20–45	NA	3 months, 0.00 6 months, 2.68 12 months, 6.25
Schuebel, 2018 [66]	Germany	NCT02449148	150	May 2015 to May 2016	RCT, single center, 3 arms, parallel	STF vs. CER vs. General diet	35–65	50.0	25–40	NA	3 months, 4.00
Mraović, 2018 [59]	Serbia	NA	240	January 2014 to May 2015	RCT, single center, 3 arms, parallel	ADF vs. CER (20%) vs. CER (50%, excluded)	20 40	100	25–44.9	≥ 35	2 months, NA 5 months, NA 10 months, 60.00
Conley, 2018 [39]	Australia	ACTRN12614000396628	24	May 2014 to April 2015	RCT, single center, 2 arms, parallel	STF vs. CER	55–75	0	≥ 30	NA	3 months, 4.16 6 months, 4.16
Oh, 2018 [60]	South Korea	NCT03652532	45	March to April and June to July 2014	RCT, single center, 4 arms, parallel	ADF vs. General diet vs. Exercise (excluded) vs. ADF + Exercise (excluded)	18–64	57.78	≥ 23	≥ 25	2 months, 22.22
Tinsley, 2017^a^ [72]	USA	NA	28	NA	RCT, single center, 2 arms, parallel	TRE vs. General diet	≥ 18	0	NA	≥ 15	2 months, 35.71
Trepanowski, 2017 [74]	USA	NCT00960505	100	October 1, 2011, to January 15, 2015	RCT, single center, 3 arms, parallel	ADF vs. CER vs. General diet	18–64	86.00	25–39.9	NA	6 months, 21.00 9 months. 31.00
Catenacci, 2016 [35]	USA	NA	29	December 2006 to May 2010	RCT, single center, 2 arms, parallel	ADF vs. CER	18–55	NA	≥ 30	≥ 35	2 months, 10.34
Moro, 2016 [58]	Italy	NA	34	NA	RCT, single center, 2 arms, parallel	TRE vs. General diet	≥ 18	0	NA	NA	2 months, 0.00
Harvie, 2013^b^ [46]	UK	ISRCTN52913838	77	September 2009 to January 2011	RCT, single center, 3 arms, parallel	CER vs. STF vs. STF + ad libitum protein and fat (excluded)	20–69	100	≥ 24	NA	3 months, 14.29
Bhutani, 2013 [32]	USA	NA	41	NA	RCT, single center, 4 arms, parallel	ADF vs. General diet vs. Excersise (excluded) vs.ADF + exercise (excluded)	25–65	96.39	30–39.9	NA	3 months, 21.95
Varady, 2013 [75]	USA	NA	32	NA	RCT, single center, 2 arms, parallel	ADF vs. General diet	35–65	73.33	20–29.9	NA	3 months, 6.25
Harvie, 2011^b^ [47]	UK	ISRCTN52913838	107	NA	RCT, single center, 2 arms, parallel	STF vs. CER	30–45	100	24–40	≥ 35	3 months, 14.02 6 months, 16.82
Stote, 2007 [69]	USA	NA	21	NA	RCT, single center, 2 arms, crossover	TRE vs. General diet	40–50	66.66	18–25	NA	2 months, 28.57

*Abbreviations: RCT* Randomized controlled trial, *TRE* Time-Restricted Eating, *eTRE* food intake restricted to the early part of the day, *mTRE* food intake restricted to the middle of the day, *ADF* Alternate day fasting, *CER* Continuous energy restriction, *STF* Short-term fasting, *NA* not available

^a/b^They are different studies, no double counting

**Fig. 2:**
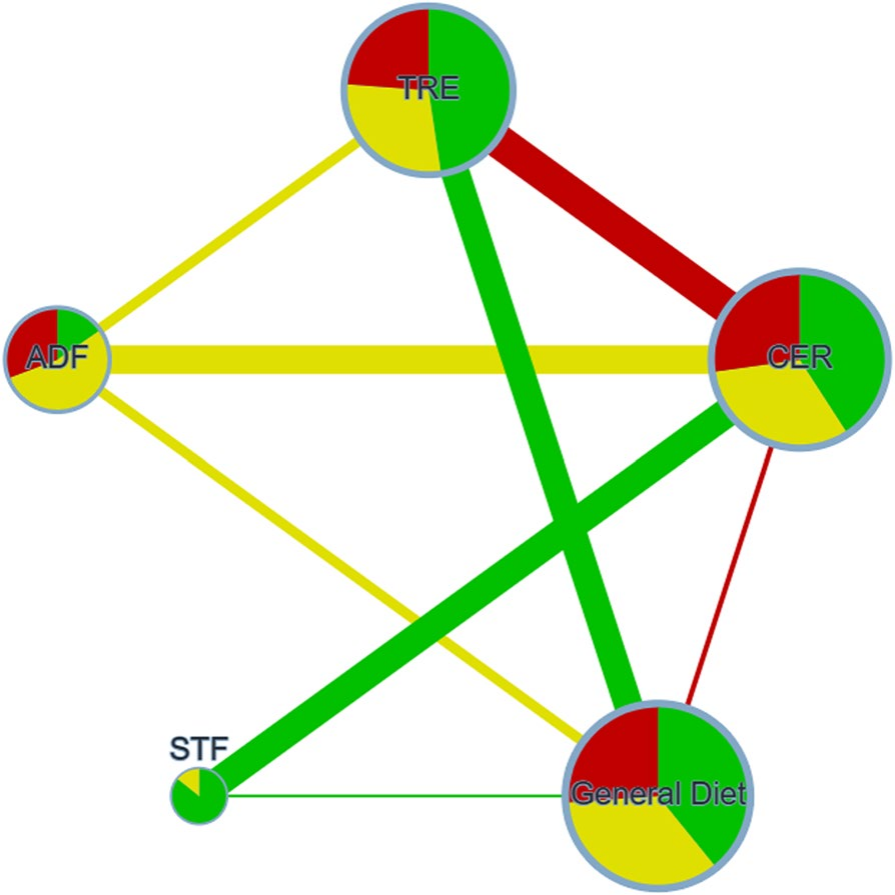
Network Plot Illustrating Body Weight Change Comparisons for Network Analysis. Each circular node represents a type of regimen, with circle size proportional to the total number of studies. Line width signifies sample size in head-to-head comparisons. Red, yellow, and green denote high, medium, and low risk of bias, respectively. ADF: Alternate Day Fasting; TRE: Time-Restricted Eating; CER: Continuous Energy Restriction; STF: Short-Term Fasting

We conducted a network comparison involving CER, STF, ADF, and TRE, establishing connections between these regimens by linking direct comparisons from the included studies, which also allow for indirect comparisons between interventions that were not directly compared (Fig. 2 and Fig. S1-2). Certainty of evidence results can be found in Fig. S6-12.

### Primary outcomes

#### Overall analysis

**Fig. 3:**
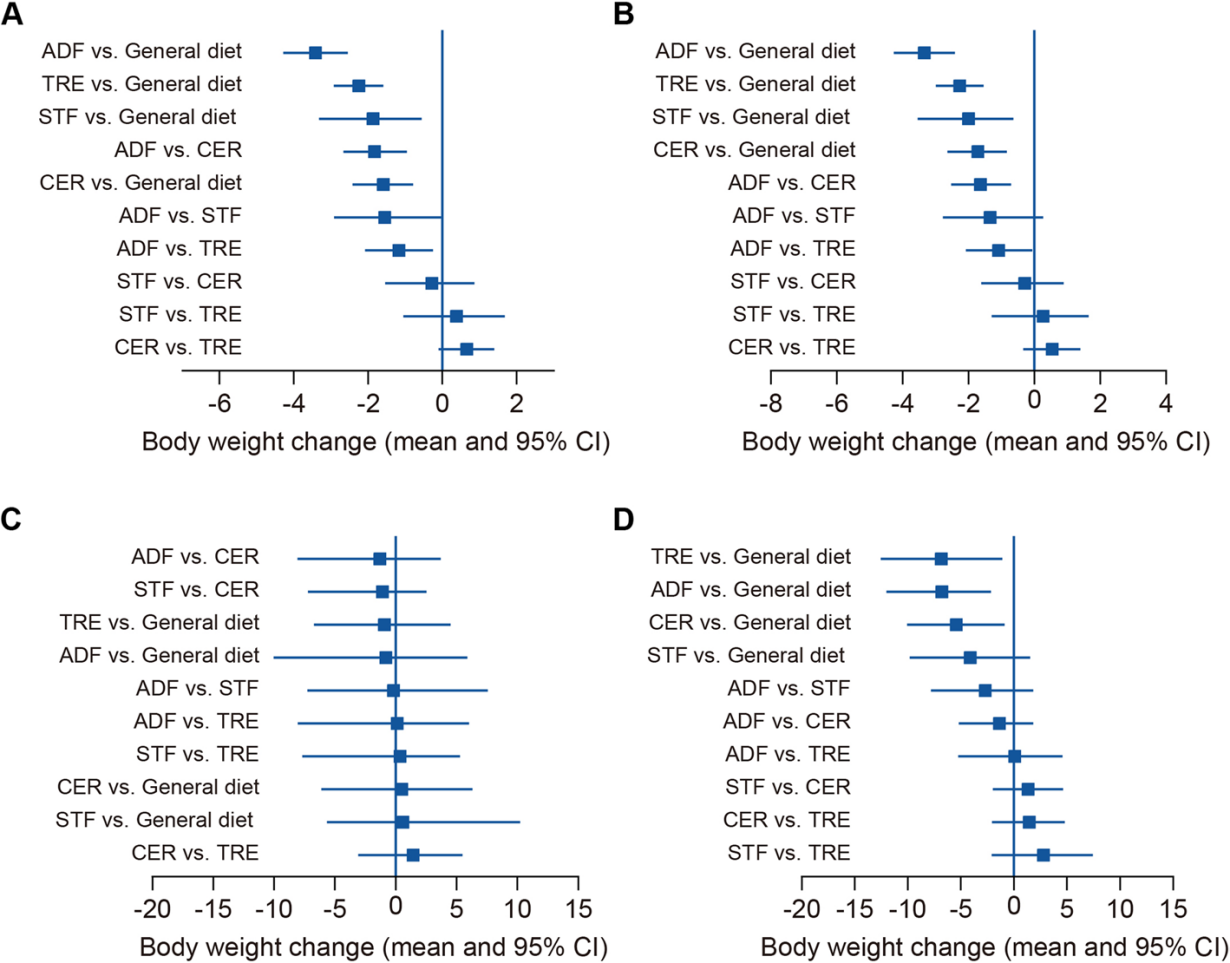
Forest plot showing comparisons among different caloric restriction regimens: **A** Overall Body Weight Change; Subgroup Network Meta-Analyses for **B** 1–3 month, **C** 4–6 month, and **D** 7–12 month periods. Regimen comparisons are sorted by efficacy. ADF: Alternate Day Fasting, TRE: Time-Restricted Eating, CER: Continuous Energy Restriction, STF: Short-Term Fasting, CI: Credible Interval

**Fig. 4:**
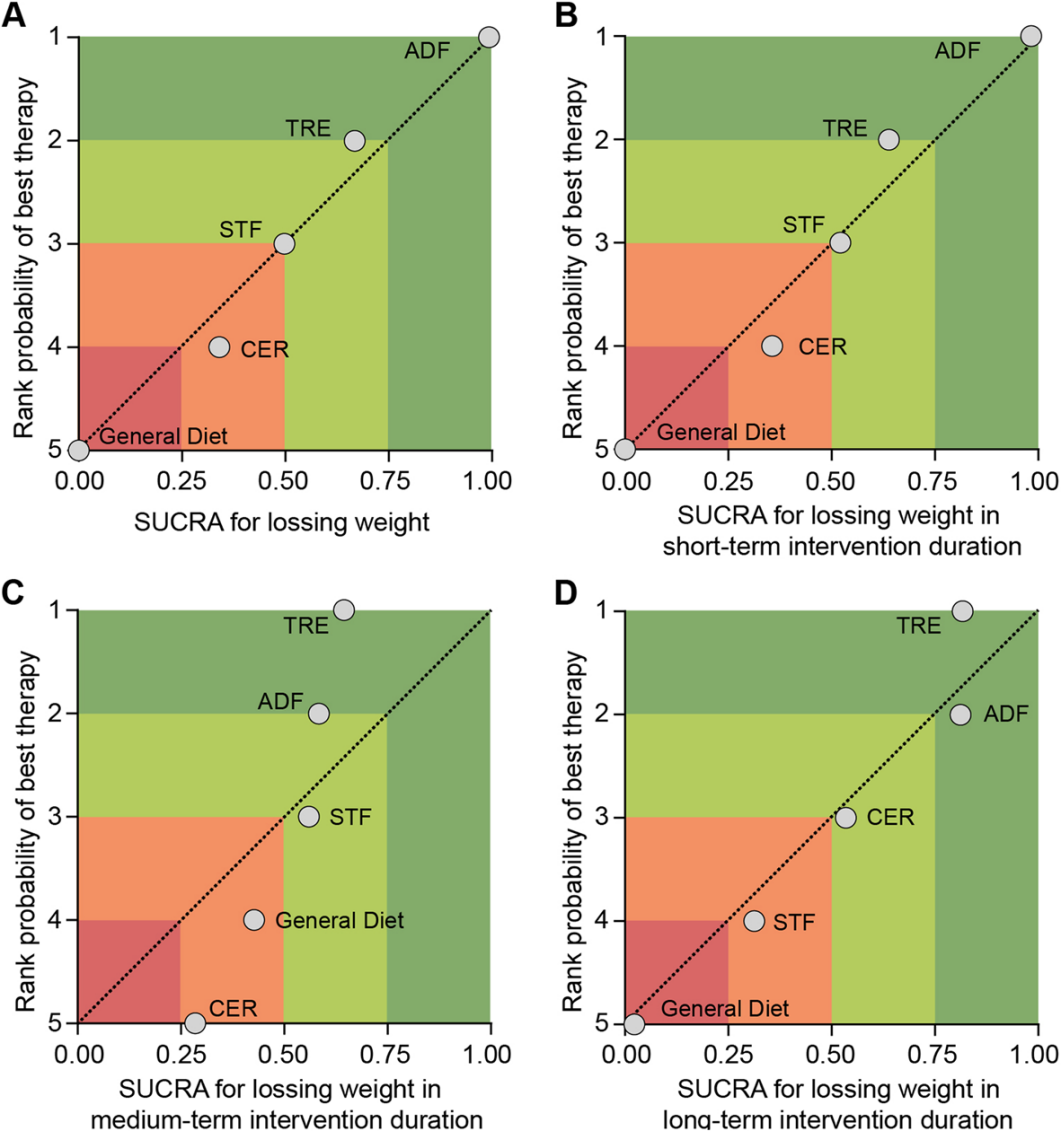
Ranking plot depicting the efficacy of caloric restriction regimens in weight loss: **A** Overall Ranking; Subgroup Analyses for **B** 1–3 month, **C** 4–6 month, and **D** 7–12 month Interventions. Strategies are plotted on the X-axis based on Surface Under the Cumulative Ranking Curve Analysis (SUCRA), with 1.00 indicating the best strategy and 0 representing the worst. The Y-axis displays the rank probability of the best strategy, with a score of 1 assigned to the top weight loss strategy. ADF: Alternate Day Fasting, TRE: Time-Restricted Eating, CER: Continuous Energy Restriction, STF: Short-Term Fasting

The network meta-analysis revealed that all caloric restriction regimens resulted in significant weight loss compared to the general diet, as illustrated in Table 2 and Fig. 3. Among these regimens, ADF demonstrated the most substantial weight loss effect (moderate-certainty evidence), surpassing TRE, STF, and CER with statistical significance (high- to low-certainty evidence). However, no significant differences were observed among TRE, STF, and CER (low- to very low-certainty evidence). Figure 3A presents the forest plot depicting the overall comparisons among the investigated regimens. This finding was further supported by the SUCRA analysis, positioning ADF in the highest position of the first quartile, as depicted in Fig. 4A.

**Table 2:** Pairwise comparisons of absolute changes in body weight

**Items**	**Comparisons**				
**Overall analysis**	**ADF**	1.83 (0.96, 2.66)*	3.42 (2.55, 4.28)*	1.55 (0.03, 2.91)*	1.17 (0.25, 2.08)*
	-1.83 (-2.66, -0.96)*	**CER**	1.59 (0.79, 2.42)*	-0.28 (-1.54, 0.86)	-0.66 (-1.4, 0.1)
	-3.42 (-4.28, -2.55)*	-1.59 (-2.42, -0.79)*	**General diet**	-1.87 (-3.32, -0.56)*	-2.25 (-2.92, -1.59)*
	-1.55 (-2.91, -0.03)*	0.28 (-0.86, 1.54)	1.87 (0.56, 3.32)*	**STF**	-0.38 (-1.68, 1.05)
	-1.17 (-2.08, -0.25)*	0.66 (-0.1, 1.4)	2.25 (1.59, 2.92)*	0.38 (-1.05, 1.68)	**TRE**
**1–3 month subgroup analysis**	**ADF**	1.63 (0.7, 2.52)*	3.34 (2.41, 4.26)*	1.34 (-0.27, 2.77)	1.08 (0.06, 2.07)*
	-1.63 (-2.52, -0.7)*	**CER**	1.71 (0.83, 2.63)*	-0.29 (-1.61, 0.89)	-0.55 (-1.4, 0.33)
	-3.34 (-4.26, -2.41)*	-1.71 (-2.63, -0.83)*	**General diet**	-2.00 (-3.54, -0.63)*	-2.26 (-2.99, -1.54)*
	-1.34 (-2.77, 0.27)	0.29 (-0.89, 1.61)	2.00 (0.63, 3.54)*	**STF**	-0.27 (-1.65, 1.29)
	-1.08 (-2.07, -0.06)*	0.55 (-0.33, 1.4)	2.26 (1.54, 2.99)*	0.27 (-1.29, 1.65)	**TRE**
**4–6 month subgroup analysis**	**ADF**	1.29 (-3.69, 8.06)	0.82 (-5.89, 10.02)	0.17 (-7.58, 7.24)	-0.12 (-6.02, 8.05)
	-1.29 (-8.06, 3.69)	**CER**	-0.49 (-6.3, 6.11)	-1.09 (-7.21, 2.52)	-1.42 (-5.48, 3.07)
	-0.82 (-10.02, 5.89)	0.49 (-6.11, 6.3)	**General diet**	-0.59 (-10.23, 5.64)	-0.92 (-6.72, 4.5)
	-0.17 (-7.24, 7.58)	1.09 (-2.52, 7.21)	0.59 (-5.64, 10.23)	**STF**	-0.35 (-5.27, 7.67)
	0.12 (-8.05, 6.02)	1.42 (-3.07, 5.48)	0.92 (-4.5, 6.72)	0.35 (-7.67, 5.27)	**TRE**
**7–12 month subgroup analysis**	**ADF**	1.36 (-1.83, 5.19)	6.80 (2.16, 12.01)*	2.69 (-1.83, 7.8)	-0.08 (-4.59, 5.23)
	-1.36 (-5.19, 1.83)	**CER**	5.43 (0.87, 10.06)	1.33 (-1.97, 4.63)	-1.45 (-4.78, 2.05)
	-6.80 (-12.01, -2.16)*	-5.43 (-10.06, -0.87)	**General diet**	-4.11 (-9.81, 1.53)	-6.86 (-12.54, -1.09)*
	-2.69 (-7.8, 1.83)	-1.33 (-4.63, 1.97)	4.11 (-1.53, 9.81)	**STF**	-2.78 (-7.43, 2.09)
	0.08 (-5.23, 4.59)	1.45 (-2.05, 4.78)	6.86 (1.09, 12.54)*	2.78 (-2.09, 7.43)	**TRE**

Comparison of the included interventions: weight change (95% CIs). Each cell gives the effect of the column-defining intervention relative to the row-defining intervention

*ADF* Alternate day fasting, *TRE* Time-Restricted Eating, *CER* Continuous energy restriction, *STF* Short-term fasting, *NA* Not available

*indicates statistical significance

#### Subgroup analyses

We present detailed mean changes in body weight along with 95% CIs for subgroup analyses based on different durations in Table 2.

The subgroup network meta-analysis conducted caloric restriction for 1–3 months revealed that all caloric restriction regimens significantly contributed to weight loss. ADF exhibited superior weight loss effects compared to CER and TRE at 1–3 months (low-certainty evidence) (Table 2), while no statistically significant differences were observed between ADF and STF (moderate-certainty evidence). Figure 3B displays the forest plot illustrating the comparisons across the investigated regimens at 1–3 months. The SUCRA analysis confirmed the findings of the 1–3 month subgroup analysis, positioning the ADF regimen in the first quartile alongside STF (Fig. 4B).

In the 4–6 month period, subgroup network meta-analysis revealed that, except for STF, all other three regimens were effective compared to the general diet (high- to low-certainty evidence) (Table 2, Fig. 3C). However, among the three regimens, no statistically significant advantage was observed (low- to very low-certainty evidence).

Due to the limited data available, the findings regarding weight changes within the 7–12 month period were inconclusive compared to previous results. However, where feasible, the SUCRA values ranked TRE as the most likely effective option for sustained weight loss exceeding three months instead of ADF (Fig. 4 C-D). Nevertheless, the 4–6 month subgroup analysis revealed no statistically significant differences between any intervention and the general diet (high- to low-certainty evidence) (Table 2), suggesting that all weight loss strategies may be associated with weight regain during this period.

**Fig. 5:**
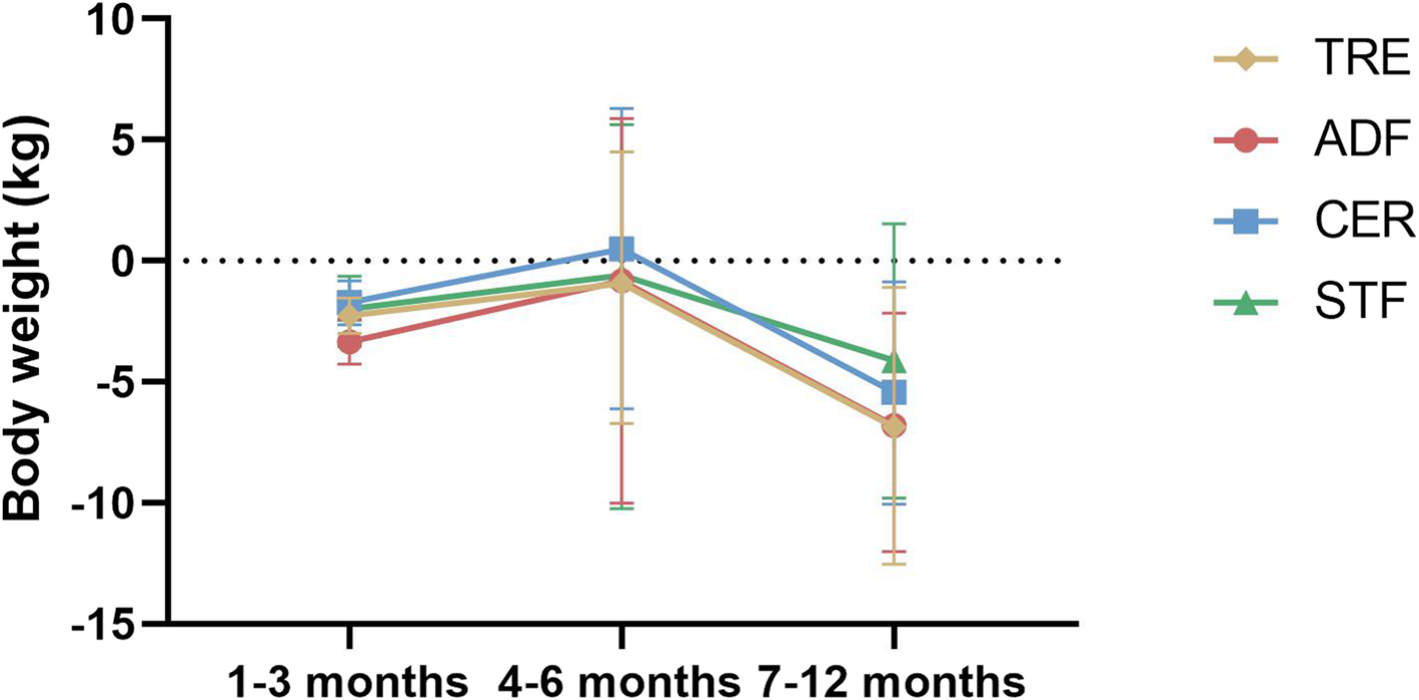
Weight change trends of four caloric restriction regimens over different durations

According to Fig. 5, all caloric restriction regimens showed weight loss effects at the 1–3 month time point compared to baseline measurements. However, in studies with durations of 4–6 months, weight regain was observed across all interventions. Notably, TRE exhibited relatively smaller magnitudes of weight regain compared to the other interventions. The results from the 7–12 month period revealed a trend toward weight loss; however, only ADF and TRE showed statistically significant results (high- to moderate-certainty evidence).

### Secondary outcomes

**Fig. 6:**
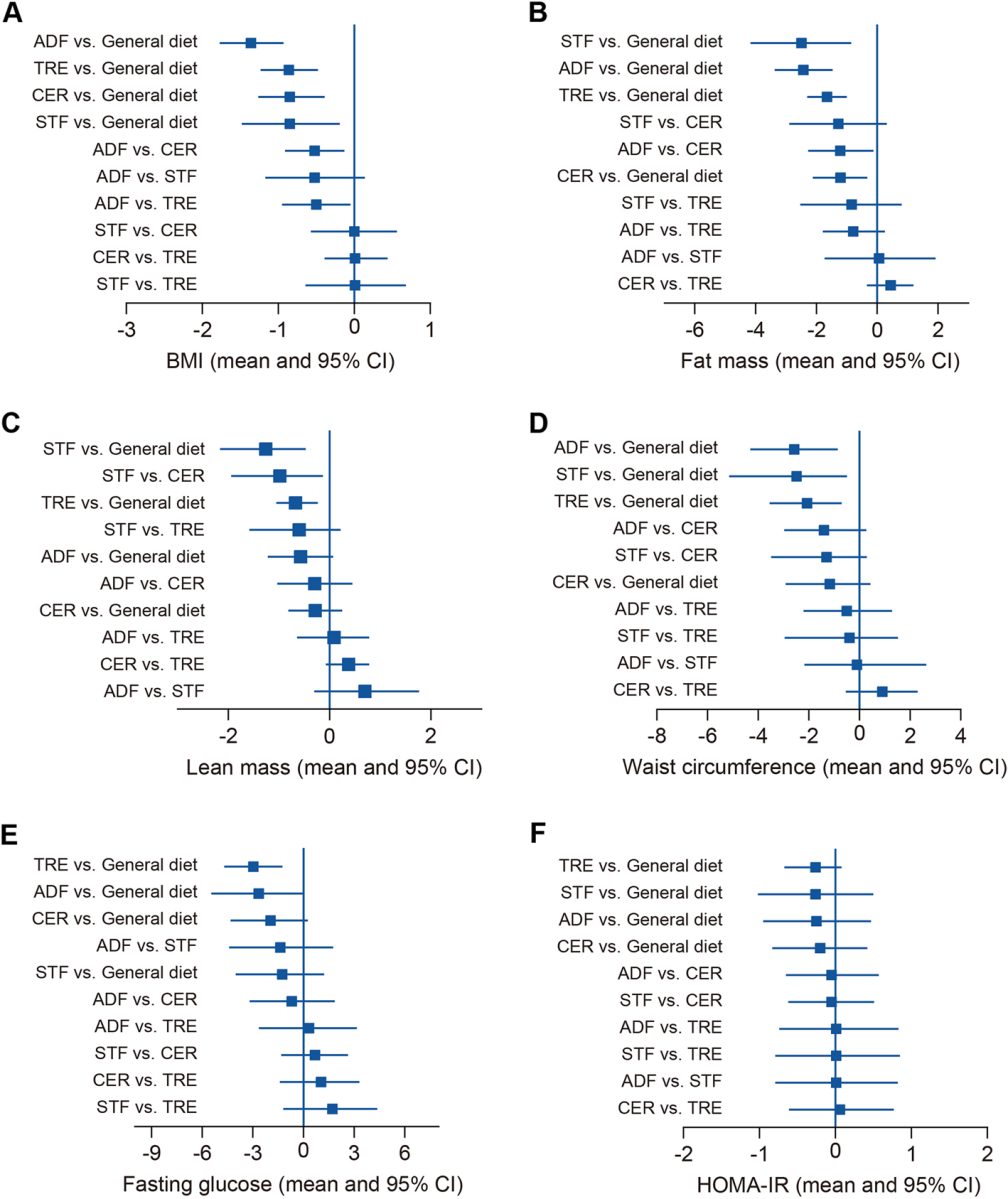
Forest plot showing overall comparisons among different caloric restriction regimens for secondary outcomes: **A** BMI; **B** Fat Mass; **C** Lean Mass; **D** Waist Circumference; **E** Fasting Glucose; and **F** HOMA-IR. Regimen comparisons are organized by efficacy. ADF: Alternate Day Fasting, TRE: Time-Restricted Eating, CER: Continuous Energy Restriction, STF: Short-Term Fasting, HOMA-IR: Homeostatic Model Assessment of Insulin Resistance, CI: Credible Interval

Detailed comparative effects with 95% CIs for the overall network meta-analysis are presented in Table S1. Subgroup network meta-analyses are provided in Tables S2-4. The forest plot illustrating the overall comparisons can be found in Fig. 6. SUCRA values are reported in Tables S5-6. Due to insufficient data availability, 4–6 month and 7–12 month subgroup analyses were partially unfeasible.

#### BMI

The overall network meta-analysis demonstrated the efficacy of all regimens in reducing BMI compared to general diet, (moderate- to very low-certainty evidence) (Fig. 6 and Table S1). In the 1–3 month period (Table S2), ADF exhibited significantly greater effectiveness compared to CER, TRE, and STF (moderate- to very low-certainty evidence), while there were no statistically significant differences observed among CER, TRE, and STF when compared to each other (high- to very low-certainty evidence). The SUCRA rankings positioned ADF first in overall and 1–3 month analyses, with TRE leading in the 7–12 months (refer to Table S5).

#### Fat mass

Both overall (Fig. 6, Table S1) and 1–3 month subgroup analyses (Table S2) demonstrated that all regimens were associated with a reduction in fat mass compared to general diet (moderate- to very low-certainty evidence). However, 4–6 month and 7–12 month subgroup analyses found no significant effectiveness for any regimen (moderate- to very low-certainty evidence) Nonetheless, TRE still emerged as relatively the most effective in 7–12 month interventions (moderate- to low-certainty evidence), as indicated by SUCRA values (Table S5).

#### Lean mass

In both the overall (Fig. 6, Table S1) and 1–3 month (Table S2) network meta-analysis, STF was associated with the most significant lean mass loss (high-certainty evidence), followed by TRE (low-certainty evidence) (Table S6) ( both are statistically significant).

#### Waist circumference

Both overall (Fig. 6, Table S1) and 1–3 month subgroup (Table S2) analyses indicated the efficacy of IER approaches, including ADF, TRE, and STF, in reducing waist circumference (moderate- to low-certainty evidence), while CER regimens did not show significance (very low-certainty evidence). ADF outperformed STF, according to SUCRA values in the 1–3 month period, while TRE remained the most effective in the 7–12 month period (Table S6). Similar to Fat mass outcomes, 4–6 month and 7–12 month analyses found no significant effectiveness for any regimen (moderate- to very low-certainty evidence).

#### Fasting glucose

Compared to the general diet, only TRE significantly reduced fasting blood glucose levels in the 1–3 month period (low-certainty evidence) (Table S2) and its highest ranking in SUCRA (Table S6). However, no regimen proved effective in the 4–6 month and 7–12 month period (moderate- to very low-certainty evidence) (Tables S3-4).

#### HOMA-IR

No significant difference was observed in reversing insulin resistance between all caloric restriction regimens and the general diet (very low-certainty evidence) (Fig. 6, Table S1-2).

#### Adverse events

Sixteen studies reported adverse events [34, 35, 38, 39, 46, 47, 55, 57, 61, 62, 66, 68, 70, 73, 75, 76]. Among them, four trials [34, 35, 57, 73] documented no adverse events throughout the intervention period. Participants in other studies experienced mild to moderate physical symptoms, which are outlined in Table 3, and no instances of serious adverse events were reported.

**Table 3:** Adverse events during the caloric restriction period

Interventions	Physical symptoms	Duration	Reference
ADF	Headaches (13%), constipation (6%)	During the first two weeks	[62, 68, 75]
STF	Hunger (54.5%), constipation (4.8%-27.3%), headaches (5%-20%), dizziness (3%-11%), mild nausea (6%), cramps (6%), fatigue (5%), lack of concentration (4%), mood swings or bad temper (3%), temporary sleep disturbance (2%), and feeling cold (NA)	Hunger and constipation improved over time	[39, 46, 70]
TRE	lack of concentration (26.3%), irritableness (10.5%), fatigue (4.4–10.5%), dizziness (5.3–8.7%), upper abdominal pain (7.3%), dyspepsia (7.3%), constipation (2.9%), nausea, headache (1.5%), dry mouth (5.3%), and diarrhea (NA)	Dizziness, nausea, headaches, and diarrhea peaked at the second week, but disappeared at the third week. Fatigue, Constipation and dry mouth did not change over the course of trial	[38, 55, 56]
CER	Dyspepsia (11.4%), dizziness (3%-7.1%), upper abdominal pain (5.7%), fatigue (5%-5.7%), mood swings or bad temper (5%), headache (2.9%-5%), feeling cold (3%), constipation (1.4%-3%), decreased appetite (2.9%), lack of concentration (2%-3%), and mild nausea (2%)	Dizziness, headache and nausea in the first four weeks	[46, 55, 66, 70]

*ADF* Alternate day fasting, *TRE* Time-Restricted Eating, *CER* Continuous energy restriction, *STF* Short-term fasting, *NA* Not available

### Quality of evidence

**Fig. 7:**
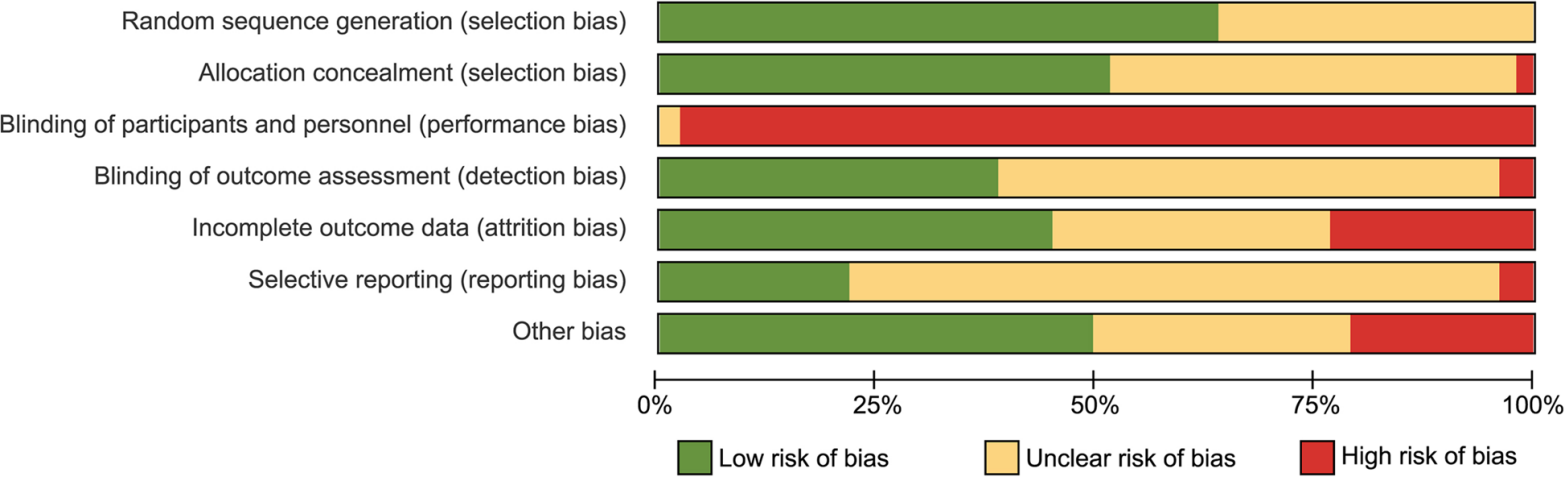
Weighted plot for the assessment of the overall risk of bias via the Cochrane Handbook’s Risk of Bias assessment tool

Figure 7 depicts the weighted plot assessing the overall risk of bias across domains, while Fig. S3 illustrates a traffic light plot. All studies were deemed to have a high risk of performance bias (100%) due to the impracticality of blinding participants to the nature of the interventions. Approximately 61.7% of the RCTs exhibited a low risk of bias concerning the randomization process. Concerns regarding bias during the allocation concealment were noted in 24 studies (51.1%),in 27 studies (57.4%) for blinding during outcome assessment, and in 29 studies (61.7%) for the process of blinding during outcome assessment. Additionally, bias concerns were identified in 37 studies (78.7%) for selective reporting and in 24 studies (51.1%) for other sources of bias. Among the 47 RCTs, 12 (25.5%) were at high risk of attribution bias related to outcome measurement, six (12.8%) due to significant dropout rates, three (6.4%) due to insufficient reporting of population dropout information, and three (6.4%) RCTs were flagged for inappropriate handling of missing data.

The certainty of evidence regarding its impact on each outcomes was assessed using the CINeMA system (Fig. S6-12).

## Discussion

In adults requiring weight loss, whether overweight or obese, the four caloric restriction regimens show high certainty of the 1–3 month period evidence for weight reduction, resulting in an average weight loss of 1.71–3.34 kg, consistent with two comprehensive systematic reviews [78, 79]. Among these regimens, ADF and TRE demonstrate relative advantages in reducing weight and BMI, with high to moderate certainty. High-quality evidence demonstrates that the STF likely lead to the most pronounced decline in lean mass while reducing weight, consistent with an umbrella review of overweight patients [80]. Moderate to low certainty evidence suggests that caloric restriction regimens may have some impact on fat mass and fasting blood glucose reduction. Limited evidence suggests that caloric restriction regimens may have little or no utility in improving insulin sensitivity, failing to provide convincing evidence of benefit.

Our study encompasses multiple outcome measures and further conducts subgroup analyses on duration while summarizing adverse reactions. ADF effectively reduces weight, BMI, fat mass, and waist circumference, consistent with several systematic reviews [25, 81, 82] and Elortegui et al.'s [82] network meta-analysis. Our findings align with Park et al.'s report [81] of improved fasting blood glucose within 6 months with lower weight regain risk compared to CER. Although our analysis did not reveal significant differences in fasting blood glucose and insulin resistance compared to the general diet group, favorable trends toward ADF were observed. This trend is consistent with Trepanowski et al.'s findings [74], which also observed improvements in plasma lipids with ADF at 6 months. Furthermore, ADF shows fewer, shorter-lasting, and less frequent side effects compared to alternative regimens.

The STF regimen, also known as the 5:2 diet in many studies [39, 48, 79, 80, 82], has gained attention as a relatively novel caloric restriction strategy for its effectiveness in weight loss over 1–3 months and the improvement of cardiovascular metabolic risk factors [70, 80]. However, our study suggests that it may not necessarily outperform other strategies. Our findings indicate that while STF achieves significant weight loss, this substantial reduction in lean mass could be considered its primary drawback, a conclusion supported by previous research [80, 83]. Moreover, STF is associated with a higher incidence of adverse effects, attributed to its characteristic prolonged fasting periods, particularly in the case of the “consecutive 2 days fasting” diet regimen, which restricts energy intake to approximately 25% for up to 48 h, explaining the prevalence of common symptoms such as hunger and constipation. Prolonged fasting is also linked to an increased risk of adverse events affecting the nervous and gastrointestinal systems (see Table 3). Despite some interveners self-suggesting a connection between these adverse reactions and a decrease in the intake of weight-loss-promoting foods, the incidence of weight regain remains elevated within STF framework [70]. Furthermore, recent basic research indicates that prolonged fasting may lead to decreased monocyte circulation and compromised immune function, thereby increasing susceptibility to infections [84]. These findings raise concerns regarding potential health implications of STF. Some studies have explored combining fasting with exercise, including resistance training, reporting the effectiveness of preserving [32, 85, 86] or even increasing [73] lean body mass while achieving weight loss simultaneously, yet the reliability of this evidence remains insufficient for conclusive determination.

TRE regimen decreased fasting blood glucose levels in the 1–3 month and 4–6 month period, albeit with lower certainty, but exhibit limited 7–12 month efficacy, correlating with trends in weight management, while their impact on improving insulin resistance remains inconclusive. Some studies indicate that IER, including TRE, can reduce HOMA-IR and fasting insulin levels in overweight individuals or those with metabolic syndrome [87–89]. Furthermore, TRE demonstrates beneficial effects on cardiovascular metabolic parameters [87, 90] and lipid profiles [76, 83, 87]. Overall, patients with higher baseline risks (e.g., diagnosed diabetes, hypertension, dyslipidemia) may derive the greater absolute benefit [79, 91, 92]. Recent high-quality research suggests that early initiation of TRE, restricting food intake to earlier parts of the day, may enhance insulin sensitivity more effectively than starting TRE from midday to evening, thereby reducing inflammation and promoting gut microbiota diversity [19], preventing excessive fat accumulation, and restoring cholesterol homeostasis through modulation of clock-related gene expression [22]. Thus, the timing and duration of fasting appear crucial for regulating glucose and lipid metabolism. However, a recent preliminary research presented at the American Heart Association’s Epidemiology and Prevention│Lifestyle and Cardiometabolic Scientific Sessions 2024 emphasized that While TRE may offer benefits in the 1–3 month period, it may not be conducive to long-term health. Individuals following a 16 + 8 TRE regimen were found to have a 96% significantly increased risk of dying from cardiovascular disease in the long term [93]. Nevertheless, considering the limitations of current evidence, further clinical research is warranted to comprehensively understand these effects.

Weight regain is a common occurrence across all four fasting regimens within the 4–6 month timeframe, with this regain nearly counteracting the initial weight loss and leading to a restoration of weight to levels close to baseline (Fig. 5). Decreased adherence to dietary protocols exert a influence on this phenomenon [94]. Complex physiological mechanisms, including various hormonal regulations and metabolic adaptations [95], further complicate the maintenance of weight loss during this period. Fasting may affect weight control by altering the composition of the gut microbiota [76, 96], while reciprocal interactions among gut microbes can also re-regulate metabolism processes [97]. Additionally, adipose tissue plays a critical role in weight regulation, influenced by factors such as cellular stress, inflammation, extracellular matrix alterations, and adiponectin secretion [97, 98]. These processes are intricately linked to the interactions between immune cells and adipocyte stroma cells [99]. Furthermore, non-physiological factors also significantly impact weight management. Emotional influences, environmental cues, and behavioral patterns may all contribute to weight regain after successful weight loss. For example, psychological stress may lead to overeating or a return to unhealthy dietary habits, thereby facilitating weight regain [100]. Environmental factors, such as the availability of high-calorie foods and sedentary lifestyles, may also present challenges to maintaining a healthy weight [101].

## Limitations

The limitations of our study are primarily influenced by the existing evidence. Firstly, concerns about the overall risk of bias arise due to the lack of adequate blinding, small sample sizes in many included studies, and high dropout rates in some studies. Secondly, the absence of data for the 4–6 month and 7–12 month periods in most studies notably reduces the synthesis accuracy of subgroup effects, resulting in lower evidence grades for these durations. Thirdly, the lack of specific BMI inclusion criteria in most studies makes subgroup analysis challenging across normal weight, overweight, and obese populations, given the diverse characteristics of the study populations. Fourthly, while SUCRA primarily provides rankings of interventions, it does not offer precise estimates of effect size or consider study quality.

## Conclusions

For adults, all four caloric restriction regimens demonstrated effective body weight loss. ADF may be the most effective in reducing body weight as well as BMI, waist circumference, fat mass, and insulin resistance. TRE appears to be relatively more effective in lowering fasting glucose, while STF may lead to the most lean mass loss.

After 1–3 months of intervention, all four caloric restriction regimens likely result in modest body weight loss, with ADF ranked first. However, by 4–6 months, varying degrees of body weight regain are observed across all regimens, with CER potentially experiencing the most significant weight rebound. Interventions lasting 7–12 months may lead to effective weight loss across all regimens. TRE may potentially rank first during both the 4–6 months and 7–12 months.

Further research is needed to focus on the long-term effects of different caloric restriction regimens on specific populations, such as those who are overweight, obese, diabetic, or have polycystic ovary syndrome. It is essential to thoroughly assess the various metabolic benefits, side effects, and the sustainability of weight loss for each regimens.

## Data Availability

Data (including the extracted contents from the searched articles) are available upon reasonable request from Dr. Jinming Huang; mail: dr.huangjinming@foxmail.com.

## Abbreviations

ADF: Alternate day fasting
TRE: Time-restricted eating
CER: Continuous energy restriction
STF: Short-term fasting
BMI: Body mass index
HOMA-IR: Homeostatic Model Assessment of Insulin Resistance
SUCRA: Surface under the cumulative ranking curve
Prbest: Probability of being the best
CINeMA: Confidence in Network Meta-Analysis
NCDs: Non-communicable diseases
IER: Intermittent energy restriction
PROSPERO: Prospective Register of Systematic Reviews
CENTRAL: Cochrane Central Register of Controlled Trials
MD: Mean differences
CIs: 95% Confidence intervals
